# Supplementary figures and images for: Identification of areas of very high biodiversity value to achieve the EU Biodiversity Strategy for 2030 key commitments
Source: PeerJ. 2020 Sep 30;8:e10067. doi: 10.7717/peerj.10067 (PMC7532765; doi:10.7717/peerj.10067)

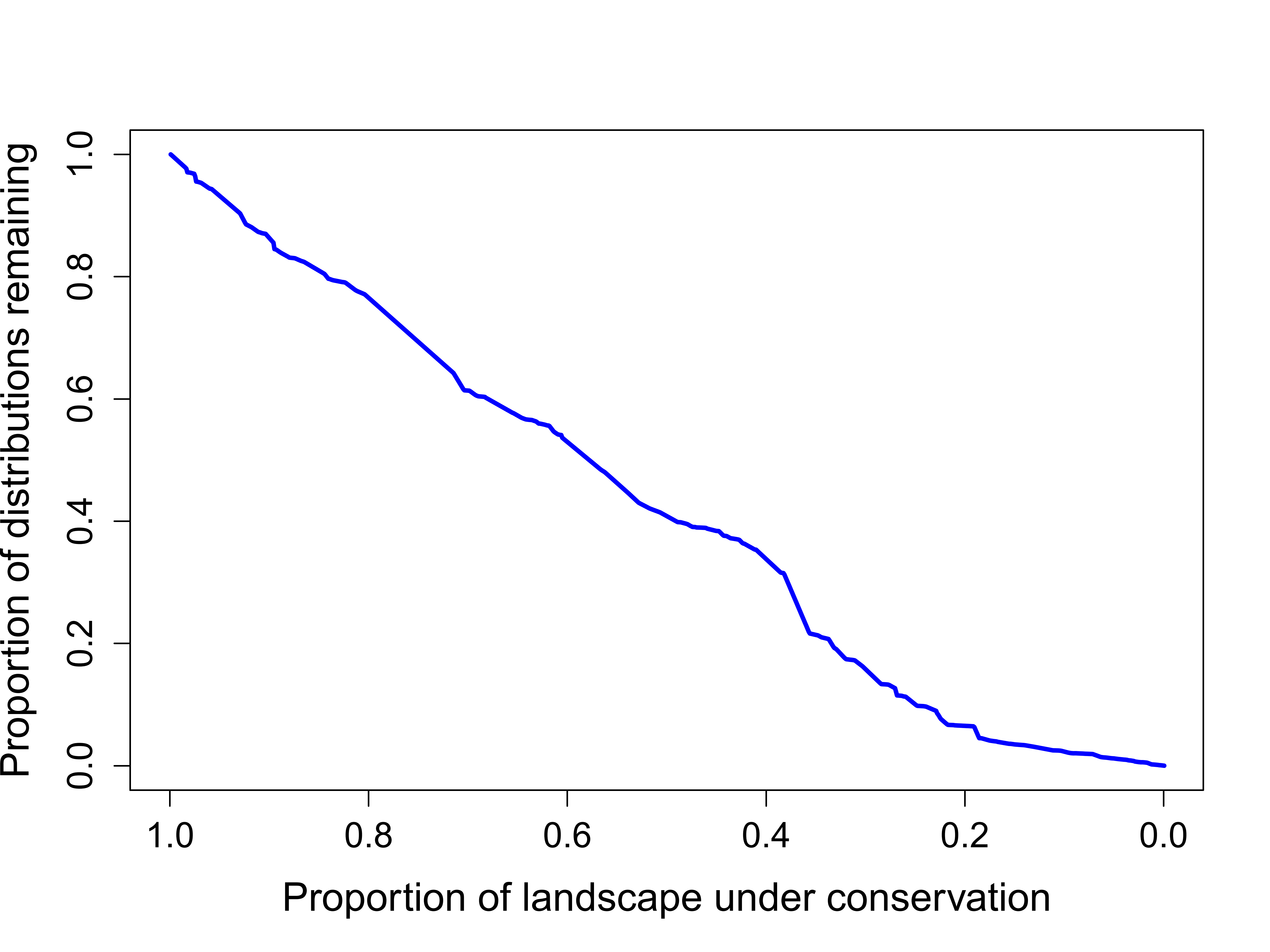

Supplement: Supplemental Information 4 — National level prioritization scenario. The curve represent mean coverage achieved across all species. [file peerj-08-10067-s004.png]

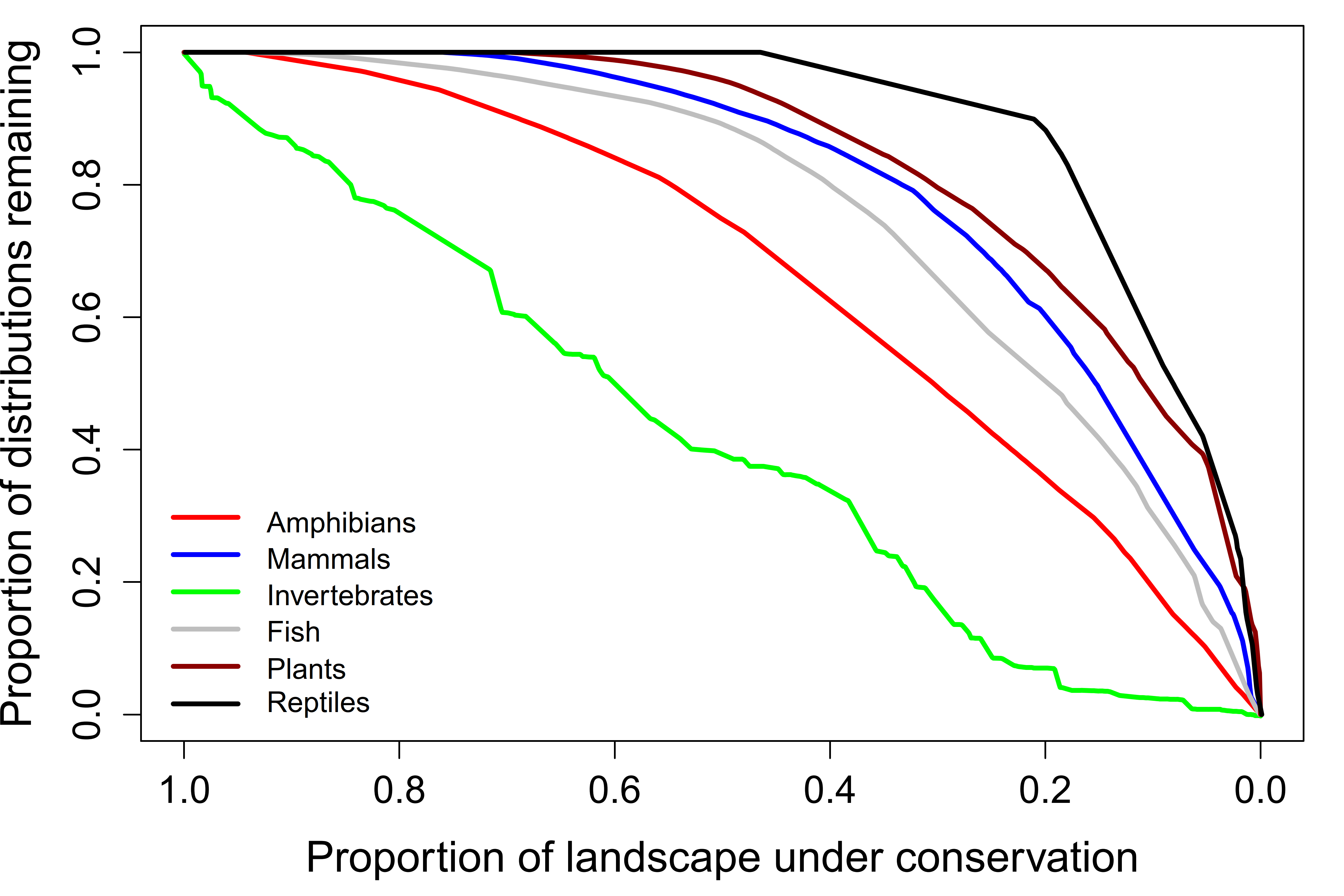

Supplement: Supplemental Information 5 — Taxonomic group level prioritization scenario. The curves represent mean coverage achieved across all species of the respective taxonomic group. [file peerj-08-10067-s005.png]

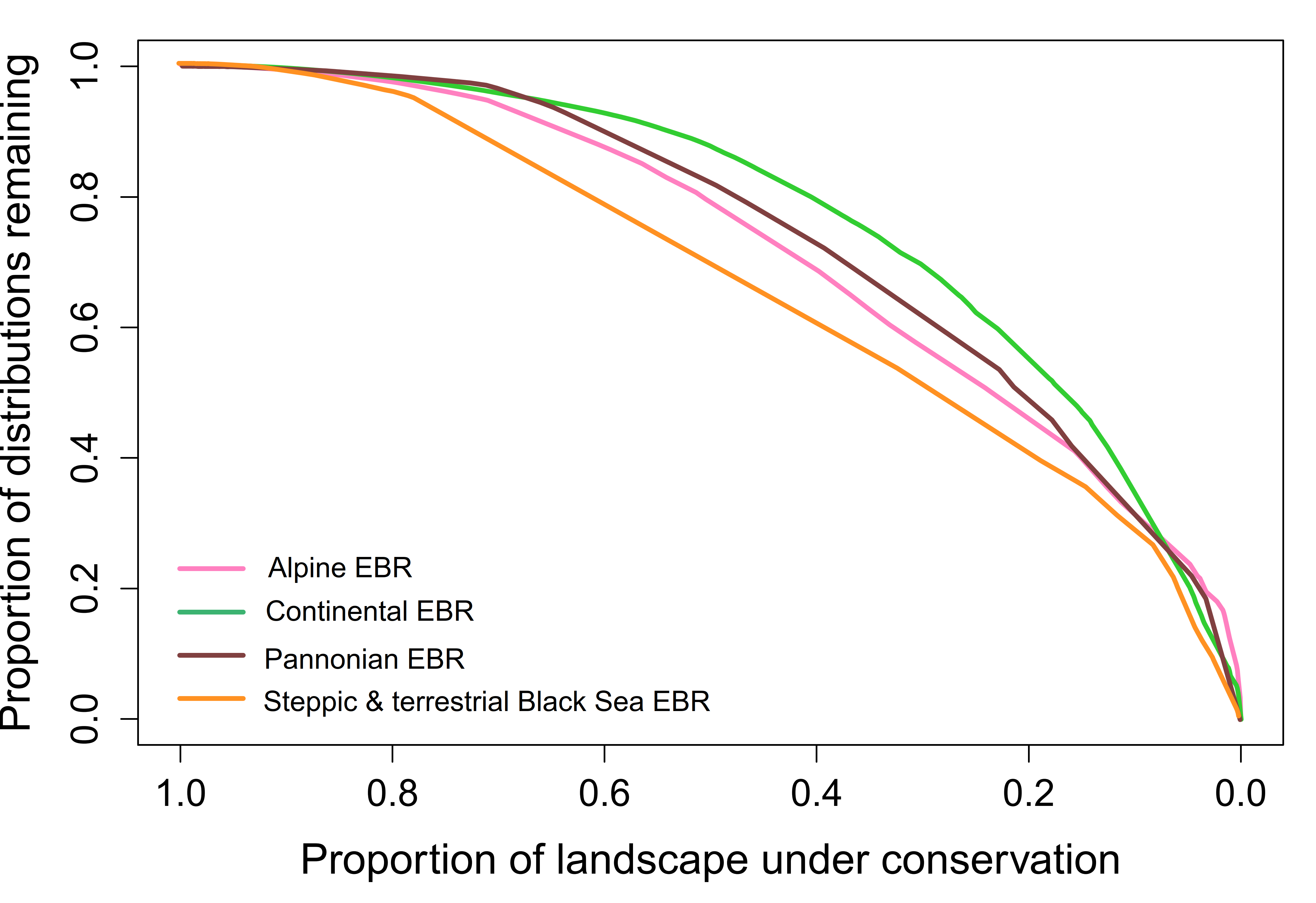

Supplement: Supplemental Information 6 — Biogeographical level prioritization scenario. The curves represent mean coverage achieved across all species. [file peerj-08-10067-s006.png]
